# Resistance to thyroid hormone beta coexisting with papillary thyroid carcinoma—two case reports of a thyroid hormone receptor beta gene mutation and a literature review

**DOI:** 10.3389/fgene.2022.1014323

**Published:** 2022-12-01

**Authors:** Yingxin Fang, Tingting Liu, Huimin Hou, Zhihong Wang, Zhongyan Shan, Yanli Cao, Xiaochun Teng

**Affiliations:** ^1^ Department of Endocrinology and Metabolism, Institute of Endocrine, NHC Key Laboratory of Diagnosis and Treatment of Thyroid Diseases, The First Hospital of China Medical University, Shenyang, China; ^2^ Department of Thyroid Surgery, The First Hospital of China Medical University, Shenyang, China

**Keywords:** resistance to thyroid hormone, papillary thyroid carcinoma, THRB gene mutation, TSH suppression therapy, thyroid hormone receptor

## Abstract

Resistance to thyroid hormone beta (RTHβ) is an autosomal dominant hereditary disorder that is difficult to diagnose because of its rarity and variable clinical features, which are caused by mutations in the thyroid hormone receptor beta (*THRB*) gene. Recent studies have indicated a close association between *THRB* mutations and human cancers, but the mechanistic role of *THRB* mutations in carcinogenesis is unknown. Herein, we report two cases of RTHβ coexisting with papillary thyroid carcinoma (PTC) and their follow-up results. Two female patients presented with elevated serum thyroid hormone levels and nonsuppressed thyrotropin (TSH). Genetic analysis showed that each patient had a *THRB* gene mutation (p.P453T and p. R320H). Based on the results of ultrasound-guided fine-needle aspiration biopsy, the thyroid nodules were suspected to be PTC. Intraoperative pathology confirmed that the two patients had PTC with multifocal carcinoma of both lobes. One patient underwent total thyroidectomy and central lymph node dissection, and the other underwent total thyroidectomy alone. Following surgery, large doses of levothyroxine were administered to suppress TSH levels and prevent recurrent or persistent disease. However, it is difficult to continually suppress TSH levels below the upper limit of the normal range. To date, the two patients have experienced no recurrence of PTC on ultrasound.

## Graphical Abstract

**Figure:**
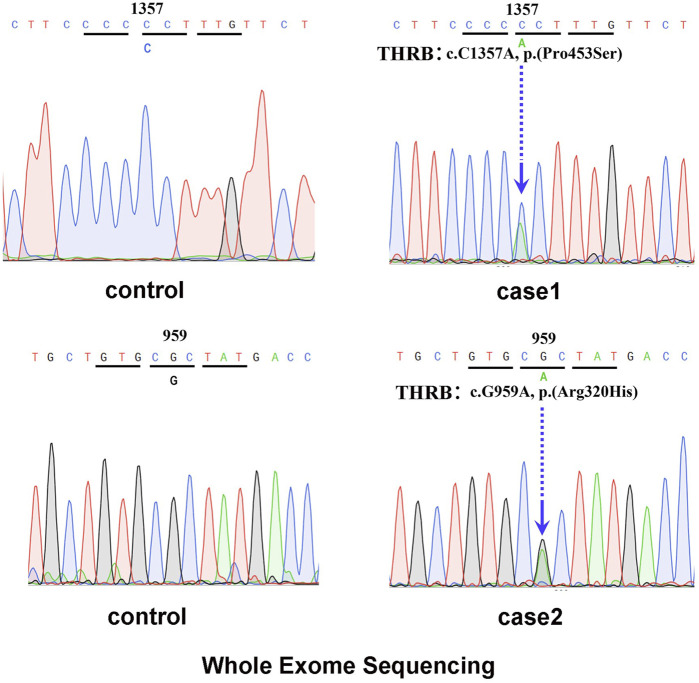
Whole exome sequencing.

## Introduction

Resistance to thyroid hormone (RTH) is a clinical syndrome defined by impaired sensitivity to thyroid hormone (TH), and its more common form, termed RTHβ, is caused by mutations in the thyroid hormone receptor beta (*THRB*) gene (Pappa and Refetoff, 2021). Surveys of 80,884 and 74,992 newborns using thyroid stimulating hormone (TSH) and thyroxine (T_4_) measurements identified 2 and 4 infants with *THRB* gene mutations, indicating a prevalence of 1 in 40,000 and 1 in 18,750 live births, respectively (Lafranchi et al., 2003; Vela et al., 2019).

RTHβ is typically characterized by elevated thyroid hormone levels and concentrations of TSH either within the normal range or mildly elevated. The clinical phenotypes of RTHβ can be highly variable and include thyroid goiter, tachycardia, and abnormal neuronal development (Ortiga-Carvalho et al., 2014).

Several studies have demonstrated that thyroid hormone receptors (TRs) are involved in human cancer (González-Sancho et al., 2003). The reduced expression of TRs, caused by hypermethylation or deletion of TR genes, found in human cancers suggests that TRs could function as tumor suppressors (Kim and Cheng, 2013). A close association between somatic mutations of TRs and human cancers further supports the notion that the loss of normal TR functioning could lead to uncontrolled growth and loss of cell differentiation (Kim and Cheng, 2013).

In the present case report, two female Chinese patients with RTHβ and papillary thyroid carcinoma (PTC) are described. Furthermore, we conduct a literature review of patients with PTC coexisting with RTHβ, and we especially discuss the follow-up of TSH suppression therapy after surgery in this rare manifestation of PTC.

### Case one

**FIGURE 1:**
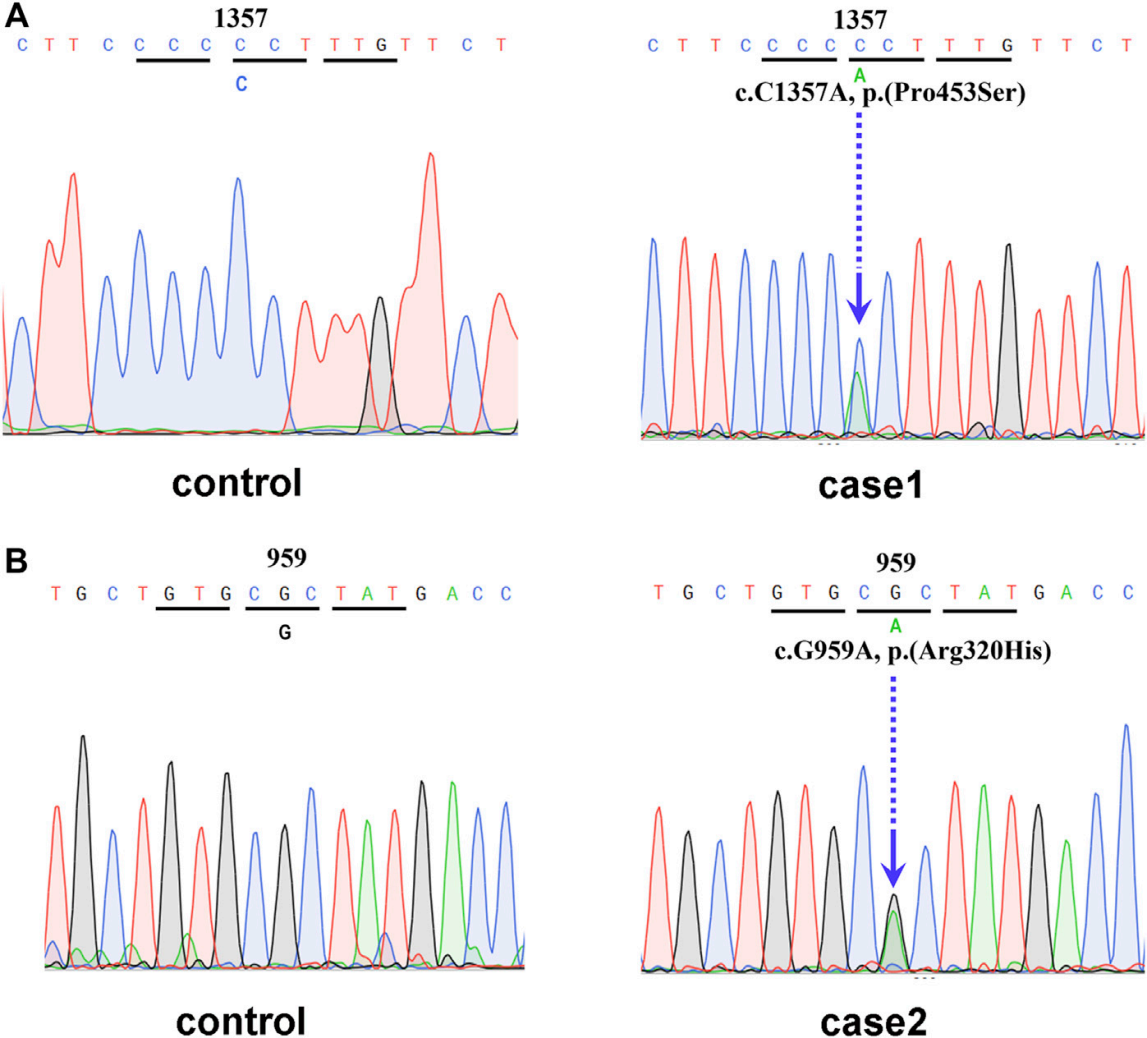
**(A)** Genetic analysis revealed a heterozygous mutation of *THRB* in exon 11 at codon 453 (P453T; c.1357C>A). **(B)** Genetic analysis revealed a heterozygous mutation of *THRB* in exon 10 at codon 320 (Arg320His, c.959G>A).

In February 2021, a 48-year-old Chinese female was referred to the Endocrinology Department due to thyroid disease. Two years prior, she noticed hyperthyroidism symptoms, such as palpitations and fatigue. She had a 3-month history of treatment with methimazole (MMI), allegedly for thyrotoxicosis. On physical examination, she presented with goiter, a heart rate of 100 beats per minute, no Graves’ ophthalmopathy and no hand tremor, although she complained of memory loss. Clinical measurements revealed out-of-range levels of circulating free thyroxine (FT_4_) and free triiodothyronine (FT_3_) in the presence of nonsuppressed TSH. Anti-thyroglobulin antibodies (TgAb) were slightly elevated, but anti-TSH receptor antibodies (TRAb) and anti-thyroid peroxidase antibodies (TPOAb) were negative (Table 1). Thyroid ultrasonography (USG) revealed multiple micronodules in the bilateral lobes that showed some suspicious features of malignancy (Thyroid Imaging Reporting and Data System (TI-RADS) grade 4c). Magnetic resonance imaging (MRI) did not show pituitary adenoma. Genetic analysis revealed a heterozygous missense mutation of *THRB* in exon 11 at codon 453 (P453T; c.1357C>A, Figure 1A). The same mutation was also detected in her mother and younger sister. Her mother also had multiple thyroid nodules (TI-RADS grade 4a), but her younger sister’s morphological features on thyroid USG were normal.

**TABLE 1:** Thyroid Indicators Measurement During Follow-Up of patient one as Related to Levothyroxine Doses.

Date	FT_3_ (pmol/L)	FT_4_ (pmol/L)	TSH (mIU/L)	TPOAb (IU/ml)	TgAb (IU/ml)	Tg (ng/ml)	LT_4_ therapy (μg/day)
01/2021	7.32 (2.43–6.01)	20.72 (9.01–19.05)	2.38 (0.35–4.94)	0.74 (0–5.61)	13.28 (0–4.11)	73.40 (1.6–59.9)	Before surgery
04/2021	3.35 (2.76–6.45)	16.75 (12–22)	68.28 (0.35–5.1)	24.10 (≤34)	81.43 (≤115)	<0.04 (3.50–77.00)	100 (1.56 μg/kg)
06/2021	4.32 (2.43–6.01)	15.13 (9.01–19.05)	48.70 (0.35–4.94)	NR	NR	NR	125 (1.95 μg/kg)
09/2021	4.42 (2.43–6.01)	20.6 (9.01–19.05)	25.40 (0.35–4.94)	6.38 (0–5.61)	2.49 (0–4.11)	<0.2 (1.6–59.9)	150 (2.34 μg/kg)
11/2021	5.77 (2.43–6.01)	25.32 (9.01–19.05)	11.49 (0.35–4.94)	NR	NR	<0.2 (1.6–59.9)	200 (3.13 μg/kg)
01/2022	7.71 (2.43–6.01)	27.86 (9.01–19.05)	3.48 (0.35–4.94)	0.85 (0–5.61)	2.31 (0–4.11)	NR	200 (3.13 μg/kg) and bromocriptine 3.75 mg/d
05/2022	6.55 (2.43–6.01)	29.13 (9.01–19.05)	5.72 (0.35–4.94	NR	NR	<0.20 (1.6–59.9)	250 (3.9 μg/kg) and bromocriptine 3.75 mg/d

FT_3_, free triiodothyronine; FT_4_, free thyroxine; TSH, thyrotropin; TPOAb, thyroid peroxidase antibody; TgAb, thyroglobulin antibody; Tg, thyroglobulin; LT_4_, levothyroxine; NR, not reported. Serum TSH, FT4, FT3, TPOAb, TgAb and Tg were tested with a chemiluminescence immunoassay (Abbott Laboratories).

In March 2021, the patient underwent total thyroidectomy and central neck lymph node dissection. Pathology revealed multifocal micropapillary thyroid carcinomas in both lobes (5 mm diameter in the right lobe, 6 mm and 7 mm diameter in the left lobe), as well as metastases of PTC in 1 out of 14 central lymph nodes. Immunohistopathological staining was positive for a *BRAF* ^V600E^ mutation. The patient was diagnosed with RTHβ and PTC (T1aN1aM0 according to version 8 of the UICC/AJCC TNM system) and was rated as low risk in accordance with the 2015 American Thyroid Association (ATA) risk of recurrence stratification system. Since there were no signs of extrathyroidal extension or distant metastases, radioactive iodine remnant ablation was not performed.

After surgery, the patient received levothyroxine (LT_4_) at 75–100 μg/day (Table 1). Three months later, her TSH level was still elevated (TSH 48.7 mIU/L), and she complained of symptoms of hypothyroidism; therefore, we increased the dose of LT_4_ to 125 μg/day. After 3 months, her TSH level decreased to 25.4 mIU/L, and most of her hypothyroid symptoms disappeared. Due to the elevated TSH level, the dose of LT_4_ was increased to 150 μg/day. After 2 months, her serum TSH level was 11.49 mIU/L. To achieve a TSH level less than 0.5 mIU/L within the first year after surgery, the dose of LT_4_ was increased to 200 μg/day. Two months after this increase, the TSH level was 3.48 mIU/L. Subsequently, LT_4_ suppression therapy was continued together with 3.75 mg/day bromocriptine. The latest thyroid function tests 15 months postsurgery revealed that the patient’s TSH, FT_4_ and FT_3_ levels were slightly increased, but she exhibited no signs of hyperthyroidism. Thyroid USG demonstrated no signs of disease persistence or recurrence, and serum Tg levels were <0.20 ng/ml.

### Case two

In November 2017, a 31-year-old Chinese female came to the clinic complaining of palpitations and dyspnea for 1.5 years that had worsened in the last 2 months. She also presented with tremor, fatigue, and poor memory. She reported a history of dyslipidemia and learning difficulties.

The laboratory examination revealed a normal TSH level despite elevated levels of thyroid hormone (Table 2). Thyroid USG showed several hypoechoic nodules with irregular borders and microcalcifications in both lobes, which indicated a grade of TI-RADS 4a. Furthermore, US-guided FNA was performed for the nodules that showed suspicious features, and the cytologic diagnosis was papillary carcinoma. *BRAF* ^V600E^ mutation analysis was positive. Pituitary MRI did not reveal any significant changes. Genetic analysis revealed a heterozygous missense mutation of *THRB* in exon 10 at codon 320 (Arg320His, c.959G>A; Figure 1B), which was also previously reported as a known mutation site in RTHβ.

**TABLE 2:** Thyroid Indicators Measurement During Follow-Up of patient Two as Related to Levothyroxine Doses.

Date	FT_3_ (2.43–6.01 pmol/L	FT_4_ (9.01–19.05 pmol/L	TSH (0.35–4.94 mIU/L	TPOAb (0–5.61 IU/ml	TgAb (0–4.11 IU/ml	Tg (1.6–59.9 ng/ml)	LT_4_ therapy (μg/day)
12/2017	6.11	23.58	1.25	0.22	11.21	NR	Before surgery
04/2018	2.19	7.79	100	0.35	9.75	NR	100 (1.35 μg/kg)
06/2018	3.61	16.21	49.11	0.38	6.71	NR	150 (2.03 μg/kg)
10/2018	4.09	26.94	26.94	3.00	4.67	3.0	100 (1.35 g/kg) thyroid tablets 160 mg/d
12/2018	8.79	20.74	0.13	NR	NR	NR	100 (1.35 μg/kg) thyroid tablets 140 mg/d
02/2019	6.31	19.29	0.343	1.36	NR	1.36	100 (1.35 μg/kg) thyroid tablets 120 mg/d
04/2019	5.86	18.8	0.217	NR	NR	1.29	200 (2.70 μg/kg)
07/2019	6.25	24.99	0.0935	1.37	2.77	2.67	200 (2.70 μg/kg)
09/2019	5.04	17.41	6.95	NR	NR	NR	237.5 (3.21 μg/kg)
12/2019	6.43	24.09	1.65	NR	2.99	2.15	250 (3.38 μg/kg)
03/2020	4.45	16.55	14.39	NR	NR	1.73	250 (3.38 μg/kg)
07/2020	3.72	11.8	69.44	NR	NR	NR	300 (4.05 μg/kg)
08/2020	5.99	21.94	2.43	NR	NR	1.71	250 (3.38 μg/kg)
04/2021	3.62	14.58	64.10	NR	NR	NR	250 (3.38 μg/kg)
05/2022	3.86	16.18	69.1	0.33	2.02	3.4	300 (4.05 μg/kg)

FT_3_, free triiodothyronine; FT_4_, free thyroxine; TSH, thyrotropin; TPOAb, thyroid peroxidase antibody; TgAb, thyroglobulin antibody; Tg, thyroglobulin; LT_4_, levothyroxine; NR, not reported; thyroid tablets, desiccated thyroid tissues made from animal thyroid glands, which contains a combination of T_4_ and T_3_. Serum TSH, FT4, FT3, TPOAb, TgAb and Tg were tested with a chemiluminescence immunoassay (Abbott Laboratories).

The patient underwent total thyroidectomy in March 2018. Histological examination showed micropapillary thyroid carcinomas (7 mm diameter in the right lobe and 2 mm diameter in the left lobe) without extrathyroidal invasion or lymph node metastasis (T1N0M0). Since the patient was evaluated as low risk for the recurrence of PTC, radioactive iodine (RAI) remnant ablation therapy was not administered. TSH suppression treatment was not initiated immediately after surgery since the patient refused to take levothyroxine. After the first month postsurgery, a thyroid function test showed a dramatic elevation in TSH concentration (>100 mIU/L). LT_4_ treatment was initiated to decrease the TSH concentration with an initial dose of 100 μg/day (Table 2). Two months later, the TSH concentration was reduced to 49.11 mU/L, and the LT_4_ dose was increased to 150 μg/day. Four months later, the thyroid function test showed a TSH concentration of 26.94 mIU/L and an elevated FT_4_ concentration of 26.936 mmol/L. Since LT_3_ was unavailable, 100 μg/day LT_4_ combined with 160 mg/day thyroid tablets derived from pig thyroid glands (containing both T_3_ and T_4_) was administered. The patient complained of obvious tachycardia. A thyroid function test showed a suppressed TSH concentration of 0.13 mIU/L, with elevated FT_4_ and FT_3_ concentrations. The dose of thyroid tablets was reduced to 120–140 mg/day. During the following 4 months, the results showed a suppressed TSH concentration of 0.22 mU/L, with normal FT_4_ and FT_3_ concentrations. However, even with β-blocker treatment, the tachycardia persisted, and the patient occasionally felt short of breath. Due to the cardiac side effects of the thyroid tablets, they were discontinued, and the dose of LT_4_ was increased to 200 μg/day. During the next 3 years, TSH was not persistently well controlled below the upper limit of the normal reference range, even though the dose of LT_4_ continued to increase (Table 2). She had no symptoms of hyperthyroidism. Her daily heart rate was approximately 60 bpm, and she was diagnosed with Grade 2 Type 1 atrioventricular block. In May 2022, considering her high TSH and Tg levels, she underwent a diagnostic ^131^I whole-body scan, which showed only normal thyroid remnants in the neck, without recurrence of thyroid carcinoma or metastasis.

## Discussion

Since Taniyama et al. reported the first case of RTHβ coexisting with PTC in 2001, 17 cases of this rare disease have been reported, listed in Table 3. As a special type of PTC, the population characteristics of patients with this disease are similar to those of patients with classical PTC. For example, it occurs in all ages from 9 to 63, and 88% of them are adults, which is consistent with the much lower incidence of PTC in children than in adults. The ratio of males to females among patients with RTHβ coexisting with PTC is 1:3.25, which is similar to the ratio of males to females (approximately 1:4.39) with PTC (LeClair et al., 2021).

**TABLE 3:** Review of cases of resistance to thyroid hormone coexisting with papillary thyroid carcinoma.

Author/Year	Country	Sex/Age	Germline mutation	Somatic mutation	PTC tumor	Therapy for PTC	Side effects of TSH suppression therapy	Thyroid function results of follow up for the last time during follow up PTC				
					Side/TNM/risk of recurrence stratification			TSH mIU/l	FT_4_ pmol/l	FT_3_ pmol/l	Tg ng/ml	Year/outcome
Taniyama et al., 2001 (Taniyama et al., 2001)	Japan	F/46	THRB	NR	Left/T1aN0M0/Low risk	Subtotal thyroidectomy	NR	NR	NR	NR	NR	NR
			R429Q									
Siristatidis et al., 2004 (Siristatidis et al., 2004)	Greece	F/26	NR	NR	NR/NR/NR	Total thyroidectomy	slight symptoms of hyperthyroid	4.5 (0.4–4.0)	NR	NR	NR	8/remission
						T_4_ 250 µg						
Kim et al., 2010 (Kim et al., 2010)	Korea	F/38	THRB	NR	Bilateral/T1aN0M0/Low risk	Total thyroidectomy	NR	15.5 (0.4–4.5)	NR	NR	NR	NR
			M310T									
Paragliola et al., 2011 (Paragliola et al., 2011)	Italy	M/48	No mutation found	NR	Left/T2N0M0/Low risk	RAI LT4 175 μg (2.18 μg/kg)	arrhythmia	82.1 (0.35–2.8)	NR	NR	5.4	10/remission
											
						Total thyroidectomy						
Paragliola et al., 2011 (Paragliola et al., 2011)	Italy	M/63	THRB	NR	unilateral/T1aN0M0/Low risk	LT_4_ (3 μg/kg)	tachycardia; insomnia anxiety	34.5 (0.35–2.8)	20.3 (10.9–19.9)	4.7 (3.5–6.5)	<0.1	6/remission
			P453T			Total thyroidectomy						
Sugita et al., 2012 (Sugita et al., 2012)	Japan	F/26	THRB	NR	NR/NR/NR		tachycardia	0.0089	30.1	8.6	NR	8/remission
						Total thyroidectomy Right radical neck dissection LT_4_ 30 μg T_3_ 500 μg	sweating					
							diarrhea					
			T334C				fatigue					
Ünlütürk et al., 2013 (Ünlütürk et al., 2013)	Turkey/USA	F/29	THRB	NR	unilateral/T1aN0M0/Low risk		tachycardia	4.1 (0.3–4.0)	NR	NR	NR	20/remission
						RAI						
			T334C			LT_4_ 150 μg Bromocriptine 2.5 mg						
Ünlütürk et al. (2013)	Turkey/USA	M/33	No mutation found	NR	unilateral/T1bN1aM0/NR	Total thyroidectomy with central lymph node dissection	NR	3.4 (0.3–4.0)	20.17 (7- 16)	5.21 (3.8–6)	0.29 (2–38)	1/remission
						LT_4_ 250 μg						
Ramos-Prol et al. (2013)	Spain	F/9	THRB	NR	Bilateral/T2N0M0/low risk	Total thyroidectomy	Without symptoms of hyperthyroidism	0.42 (0.35–4.94)	16.9 (9- 19)	NR	<0.20	10/remission
						TRIAC 1.4 mg						
			R243W			LT_4_ 200 μg (4.3 μg/kg)						
Vinagre et al. (2014)	Portugal	F/19	THRB	BRAF V600E (+)N-RAS, H-RAS	V600E (+)N-RAS, H-RAS	left/T1aN0M0/low risk	Total thyroidectomy	tremors; weight loss	5 (0.4–4.4)	45 (11.45–22.65)	NR	1.8
								4/cervical lymph node metastasis				
						Central lymph node dissection						
						RAI	irritability; sudation					
			R320C	TERT (-)		LT_4_ 300 μg (5.35 μg/kg)						
Aoyama et al. (2015)	Japan	F/54	THRB	NR	Bilateral/T1aN0M0/low risk	Total thyroidectomy and cervical lymph node dissectionLT_4_ 350 μg	Without symptoms of hyperthyroidism	0.45 (0.5–4.3)	57.9 (9.0–21.9)	9.24 (3.5–6.3)	NR	2.25/remission
			P453S									
Karakose et al. (2015)	Turkey	F/56	THRB	NR	Right/T1aN0M0/low risk	Total thyroidectomy	Symptoms of hyperthyroidism	23.6 (0.55–4.78)	14.3 (9.5–19.6)	NR	NR	0.33/remission
			A234D			LT_4_ 150 μg triiodotironin 50 μg						
Karakose et al. (2015)	Turkey	M/33	THRB	NegativeBRAF	left and isthmus/T1aN0M0/low risk	Total thyroidectomy	NR	150 (0.55–4.78)	16.2 (9.5–19.6)	NR	NR	0.17/remission
						RAI						
			A234D	V600E		LT_4_ 100 μg						
Igata et al. (2016)	Japan	F/26	THRB	NR	Right/T2bN1bM0/intermediate risk	Total thyroidectomy and neck lymph nodes dissection	Without symptoms of hyperthyroidism	4 (9-19)	NR	NR	Basal 6; TSH-stimulated 12–20	12/remission
			P452L			Thyroxine 500 μg						
Xing et al. (2017)	China	F/11	THRB	BRAF	Bilateral/T1aN1bM0/Low risk	Total thyroidectomy, and lymph node dissection of the left side of the neck	Without symptoms of hyperthyroidism	6.80 (0.34–5.6)	15.97 (7.86–14.1)	5.79 (3.8–6.0)	NR	0.5/remission
						RAI						
						LT_4_ 150 μg						
			L454FS	V600E (+)		TH tablets 50mg, Bromocriptine 3.75 mg						
Current	China	F/48	THRB	BRAF	Bilateral/T1aN1aM0/Low risk	Total thyroidectomy and central lymph node dissection	Without symptoms of hyperthyroidism	5.72 (0.35–4.94)	29.13 (9.01–19.05)	6.55 (2.43–6.01)	0.20 (1.6–59)	1.25/remission
						LT_4_ 250 μg (3.9 μg/kg),Bromocriptine						
			P453T	V600E (+)		3.75 mg						
Current	China	F/31	THRB	BRAF	Bilateral/T1aN0M0/Low risk	Total thyroidectomy	Without symptoms of hyperthyroidism	69.1 (0.35–4.94)	16.18 (9.01–19.05)	3.86 (2.43–6.01)	3.4 (1.6–59)	4.5/remission
			R320H	V600E (+)		LT_4_ 250 μg (3.33 μg/kg)						

ATD, anti-thyroid drug; NR, not reported; LT_4_, levothyroxine; TSH, thyrotropin; FT_3_, free triiodothyronine; FT_4_, free thyroxine; Tg, thyroglobulin; TRIAC, triiodothyroacetic acid; TH tablets, derived from pig thyroid gland (containing T_3_ and T_4_); PTC, papillary thyroid carcinoma; TNM was according to the version 8 of the UICC/AJCC TNM system, and the risk of recurrence stratification was in accordance with the 2015 American Thyroid Association (ATA) risk of recurrence stratification system. RAI, radioactive iodine remnant ablation.

Usually, PTC in children and adolescents strongly tends to be multifocal and aggressive and easily invades outside the thyroid capsule, directly involving the recurrent laryngeal nerve, trachea, blood vessels and esophagus. Pediatric papillary thyroid carcinoma has a higher probability of lymph node metastasis and distant metastasis at the time of diagnosis, reaching up to 40%–80% (Piciu et al., 2012; Almosallam et al., 2020). In our literature review of 17 cases, two were children. Consistent with the findings of pediatric PTC, both had bilateral and multifocal thyroid cancer, and one had central lymph node metastases at the time of diagnosis (Ramos-Prol et al., 2013; Xing et al., 2017).

Among the 17 patients with RTHβ and PTC, two patients were diagnosed with RTHβ due to a refractory increase in TSH after total thyroidectomy, but the sizes, number, and lymph node metastasis of the thyroid tumors were not described. After excluding these two cases, 11 of the remaining 15 patients had papillary thyroid microcarcinoma (PTMC), 4 patients had lymph node metastases at diagnosis, and 1 patient had lymph node metastases 4 years after surgery; thus, the proportion of lymph node metastases was 33.3%. Postoperative risk of recurrence assessment showed that 13 cases were at low risk, 1 case was at intermediate risk, and the risk of the last case was not available. However, no distant metastases were reported.

Of the 15 cases, histopathologic variants of thyroid carcinoma associated with more unfavorable outcomes (e.g., tall cell, columnar cell, and hobnail variants of PTC) were not reported, and more favorable outcomes, such as a follicular variant of PTC, were found in 2 cases. *BRAF* testing was performed in only 5 of the 15 patients, and the *BRAF* ^V600E^ mutation was found in 4 of those 5 patients (80%). Other molecular markers (including *THRB, N-RAS, H-RAS,* and *TERT*) were detected in only 1 patient, but no mutations were found.

A challenging issue in patients with RTHβ and PTC is the determination of the optimal surgical treatment strategies for the tumors. Table 3 shows that 7 of the 15 patients (47%) had bilateral and multifocal PTC. The review of the literature revealed that 16 of the 17 patients underwent total thyroidectomy; among them, only 1 patient developed central lymph node metastasis. The ATA guidelines state that total thyroidectomy and central lymph node dissection can help prevent tumor persistence and recurrence in patients with differentiated thyroid cancer (DTC) (Haugen et al., 2015). Table 3 shows that 10 patients who had no lymph node metastases at the time of diagnosis did not undergo prophylactic central lymph node dissection. Among them, 9 adult patients had no recurrence or metastasis, whereas one 19-year-old adolescent girl developed central lymph node metastasis after total thyroidectomy. Therefore, prophylactic central lymph node dissection would be recommended in adolescents with this rare disease. However, for adults, it is not yet clear if it is necessary to perform prophylactic central lymph node dissection.

Another issue for patients with RTHβ and PTC is determining whether to implement RAI therapy after total thyroidectomy. The ATA guidelines indicate that ^131^I adjuvant therapy can effectively improve overall survival (OS) and disease-free survival (DFS) in DTC patients with a high risk of recurrence. For intermediate-risk patients, the overall benefit of ^131^I adjuvant therapy is still controversial, and low-risk patients do not exhibit significantly improved OS or DFS (Haugen et al., 2015). Among the 17 patients, one child with intermediate risk received RAI, and one adult with intermediate risk did not. No recurrence or lymph node metastasis was found in the 2 patients during the follow-up period. However, due to the small number of intermediate-risk cases, whether intermediate-risk patients need RAI therapy is unclear. All 13 patients at low risk did not have tumor recurrence or lymph node metastasis during follow-up, regardless of whether they received radioiodine therapy.

Another challenging issue in patients with RTHβ and PTC is TSH suppression therapy. Usually, the dose of LT_4_ in TSH suppression therapy for classical PTC is between 1.5 and 2.5 μg/kg/day, but it is difficult to suppress TSH below the upper limit of the normal reference range in patients with RTHβ and PTC, even with very large doses of LT_4_ (Table 3). Four patients were treated with triiodothyronine or thyroid tablets (a mixture of T_4_ and T_3_) to assist TSH suppression, but their cardiac side effects were difficult to overcome. It was demonstrated that 3,5,3’ triiodothyroacetic acid (TRIAC) has a higher affinity for TRβ1 than T_3_, which may suppress TSH without causing as severe a peripheral tissue effect. LT_4_ combined with TRIAC was used in 1 of the 17 cases (Ramos-Prol et al., 2013), and TSH was well inhibited without cardiac side effects (Beck-Peccoz et al., 1983; Salmela et al., 1988). Although bromocriptine also had a suppressive effect on serum TSH, the inhibition did not appear to be significant in our patient. The safe range at which serum TSH levels should be controlled to not only prevent the recurrence of PTC but also avoid the probable occurrence of TSH tumors caused by long-term high TSH levels requires further study.

Confusingly, although a very large dose of LT_4_ was administered, the serum T_3_ and T_4_ concentrations in the patients with RTHβ and PTC were disproportionately elevated or within the normal reference range (Table 3). LT_4_ is the same as the thyroxine naturally secreted by the thyroid gland. Usually, the intake of large doses of LT_4_ can cause an increase in serum thyroxine levels. This prompted us to think about potential alterations in thyroxine metabolism in patients with RTHβ and PTC after total thyroidectomy, such as a shortened half-life of thyroxine or abnormal transformation from thyroxine to the other thyroid metabolites, but these hypotheses require further study.

The concomitant presence of RTHβ and PTC raises the question of whether patients with RTHβ are at an increased risk for thyroid cancer. The precise contribution of RTHβ to thyroid tumorigenesis is not fully understood, but there is evidence to suggest that it may play a contributory role. First, it has been postulated that TSH is a growth factor, and the continuous stimulation of TSH can promote the development of nodules and thyroid cancer. Second, the TRβ mutation itself can also be somewhat pro-oncogenic (Kim et al., 2010). The levels of TRβ mRNA were significantly higher in normal and hyperplastic thyroid tissues than in neoplastic thyroid tissues (Wallin et al., 1992). Sequencing of TRβ1 and TRα1 cDNAs cloned from 16 papillary thyroid cancers revealed that mutations affected receptor amino acid sequences in 93.75% and 62.5% of cases, respectively. In contrast, no mutations were found in healthy thyroid controls (Puzianowska-Kuznicka et al., 2002). In addition, mice that harbored a knock-in mutant TRβ gene (TRβ PV mutant) spontaneously developed thyroid cancer and distant metastasis similar to human follicular thyroid cancer (Furuya et al., 2006). Further study indicated that the more aggressive thyroid tumor progression in *Thrb* ^PV/PV^ mice was due not only to the loss of tumor suppressor functions of TR *via* mutation but also, importantly, to gain-of-function in the oncogenic activities of PV to drive thyroid carcinogenesis. This identifies a novel mechanism by which a mutated TRβ evolves with an oncogenic advantage to promote thyroid carcinogenesis (Lu and Cheng, 2011). Third, the *BRAF* ^V600E^ mutation, identified in between 29 and 83% of PTC cases (Trovisco et al., 2007; Xing et al., 2017) and considered an early or initiating event in PTC, is highly expressed in patients with RTH coexisting with PTC who have received *BRAF* testing (Table 3). Whether the *BRAF* ^V600E^ mutation and RTHβ are jointly involved in the occurrence of PTC deserves further study.

In conclusion, our literature review of the 17 cases of RTHβ coexisting with PTC revealed that patients with this rare disease seem to have a good prognosis. However, due to the limited number of patients and the short-term follow-up for many of them, the recurrence rate of this rare disease may be higher than that reported here. Unsuppressed or increased serum TSH levels in the background of RTHβ are associated with an increased risk of PTC recurrence and metastasis. Therefore, total thyroidectomy is recommended for adult patients, and total thyroidectomy and prophylactic central lymph node dissection are recommended for children and adolescents. Close follow-up of the current cases is needed, and benign or malignant thyroid nodules should be evaluated during the follow-up of patients with RTHβ.

## Data Availability

The original contributions presented in the study are included in the article/Supplementary Material, further inquiries can be directed to the corresponding authors.

## Abbreviations

ATA, American Thyroid Association; DTC, differentiated thyroid cancer; DFS, disease-free survival; FT_4_, free thyroxine; FT_3_, free triiodothyronine; FNA, fine needle aspiration; LT_4_, levothyroxine; LT_3_, triiodothyronine; MMI, methimazole; MRI, magnetic resonance imaging; OS, overall survival; PTMC, papillary thyroid microcarcinoma; PTC, papillary thyroid carcinoma; RTH, resistance to thyroid hormone; RAI, radioactive iodine; THRβ, thyroid hormone receptor beta; TSH, thyrotropin; TH, thyroid hormone; TRs, thyroid hormone receptors; TgAb, anti-thyroglobulin antibodies; TRAb, anti-TSH receptor antibodies; TPOAb, anti-thyroid peroxidase antibodies; TRIAC, 3,5,3′ triiodothyroacetic acid; USG, ultrasonography.
